# GH Responsiveness Is not Correlated to *IGF1* P2 Promoter Methylation in Children With Turner Syndrome, GHD and SGA Short Stature

**DOI:** 10.3389/fendo.2022.897897

**Published:** 2022-06-13

**Authors:** Anja Apel, Daniel I. Iliev, Christina Urban, Karin Weber, Roland Schweizer, Gunnar Blumenstock, Sarah Pasche, Vanessa Nieratschker, Gerhard Binder

**Affiliations:** ^1^ Pediatric Endocrinology, University Children`s Hospital Tübingen, Tübingen, Germany; ^2^ Department of Clinical Epidemiology and Applied Biometry, University of Tübingen, Tübingen, Germany; ^3^ Department of Psychiatry and Psychotherapy, Tübingen Center for Mental Health, University Hospital Tübingen, Tübingen, Germany

**Keywords:** IGF-1, promotor methylation, GH response, prediction, short stature, GH treatment

## Abstract

**Background:**

The methylation of *IGF1* promoter P2 was reported to negatively correlate with serum IGF-1 concentration and rhGH treatment response in children with idiopathic short stature. These findings have not yet been confirmed.

**Objective:**

This study aimed to determine *IGF1* promoter P2 methylation in short children treated with rhGH and correlate clinical parameters with the methylation status. In addition, long-term stability of methylation during rhGH treatment was studied.

**Design:**

This was a single tertiary center study analyzing clinical GH response and IGF-1 serum concentration changes in patients with GHD (n=40), SGA short stature (n=36), and Turner syndrome (n=16) treated with rhGH. Data were correlated to the methylation of two cytosine residues (-137, +97) of the P2 promoter of *IGF1* in blood cells measured by pyrosequencing in 443 patient samples.

**Results:**

Basal and stimulated IGF-1 concentrations, first year increment in height velocity and studentized residuals of a prediction model did not correlate to the methylation of -137 und +97 in *IGF1* P2 promoter. The methylation of these two sites was relatively stable during treatment.

**Conclusions:**

This study did not confirm *IGF1* P2 promotor being a major epigenetic locus for GH responsiveness in patients treated with a normal dose of rhGH. Additional studies are warranted.

## Introduction

Treatment of short stature with recombinant human GH (rhGH) is well established, but response to and efficacy of the treatment varies from indication to indication and from patient to patient (1). Pharmacogenetics of the GH/IGF-1 axis attempts to explain some of the interindividual variability (2).

The growth hormone receptor (GHR) plays a key role in GH signaling and exists polymorphically in a full-length and an exon 3-deleted form (3). This is one of the most studied gene polymorphisms and has been presented in many association studies, occasionally yielding contradictory results (4, 5). In principle, *in-vitro* studies support better signal transduction by the exon 3-deleted receptor (4). Two metanalyses reported an advantage for carriers of one or two exon 3-deleted GHR alleles in terms of initial growth response to rhGH (6, 7). In addition, a variant of the IGFBP-3 promoter (-202 A/C) has been reported to be associated with serum IGFBP-3 level and response to rhGH (8, 9). Furthermore, a polymorphic (CA)n repeat 1kb upstream of *IGF1* was reported to correlate with first year height velocity in children with severe GHD (10).

Recently, the methylation of the *IGF1* promoter P2 has been linked to the response to rhGH treatment in children with idiopathic short stature (ISS) by a French study group (11). In principle, an increased amount of 5-methyl cytosine in the promoter region of a gene can cause partial or complete inactivation of gene expression (12). Methylation of CpG islands in the promoter region of *IGF1* could therefore affect IGF-1 production and thus responsiveness to GH (13). Ouni et al. reported that the methylation of specific cytosines in the *IGF1* P2 promoter in blood leucocytes negatively correlates with the transcriptional activity of *IGF1* and may explain approximately 25% of variability in responsiveness to rhGH in children with idiopathic short stature (11, 14, 15). These results have not been confirmed so far.

The aim of this project was to analyze the clinical relevance of *IGF1* promoter P2 methylation at DNA positions -137 and +97 in a cohort of children with GHD, Turner syndrome and SGA short stature treated with rhGH. In addition, we investigated the stability of the specific DNA methylation sites in blood of the patients longitudinally.

## Study Participants

A total of 92 patients with short stature and rhGH treatment donated residual blood from routine examinations for research in the Pediatric Endocrinology section of the University Children`s Hospital Tübingen since 2015. They were diagnosed with GHD (n=40), short stature after being born small for gestational age (SGA short stature) (n=36), or Turner syndrome (n=16). Height was measured using a wall-mounted stadiometer system Dr. Keller II (Längenmeßtechnik GmbH Limbach, Germany). Growth hormone deficiency was diagnosed in children with growth retardation, delayed bone age, pathologically low IGF-1 and two pathological GH stimulation tests. SGA short stature was defined by birth weight or birth length < -2 SDS and height < -2.5 SDS at 4 years of age after exclusion of other specific diagnoses. Turner syndrome was diagnosed in short girls with a karyotype that partially or completely lacked one sex chromosome. Bone age was determined using the atlas by Greulich and Pyle (16). For correlation with promoter P2 methylation, height velocities were excluded from children who were < 4 years of age at the start of rhGH treatment.

Both, the establishment of the biobank (residual blood) and this specific study were independently approved by the Ethics Committee of the Medical Faculty of the University of Tübingen (259/2014BO1 and 050/2020BO2). The study was conducted in accordance with the Declaration of Helsinki of the World Medical Association. All patients´ caregivers provided written informed consent.

## Materials and Methods

### DNA Isolation From Blood Samples

Venous blood was drawn from the studied patients at each routine visit for diagnostic purposes.

The remaining EDTA blood was stored at -20°C until further analysis. DNA was extracted using NucleoSpin Blood L (Midi-Kit Macherey&Nagel, Düren, Germany).

### Bisulfite Conversion

Bisulfite conversion was performed using the EpiTect Fast Bisulfite Conversion Kit (Qiagen, Hilden, Germany) according to manufacturer´s instruction. Briefly, 100 ng DNA was mixed with bisulfite solution and DNA Protect buffer, converted, purified with spin columns, and later eluted using 20 µl EB buffer. The concentration of bisulfite-converted DNA was determined.

### PCR and Pyrosequencing of *IGF1P2*

Converted patient samples, control samples (Qiagen; 0%, 50%, 100% methylated human control DNA and H_2_O), and three converted internal control DNA samples were amplified with appropriate primers covering the two CpG islands of the *IGF1* P2 promoter: IGF-1P2_232-108-FB/IGF-1P2_232-108_RN and IGF-1P2_77-97_FB/IGF-1P2_77-97_R. PCR amplification was performed using HotStar Taq DNA polymerase (Qiagen, Hilden, Germany) and controlled by agarose gel electrophoresis. Primers were used as previously reported (11).

Pyrosequencing was performed using Qiagen PyroMark Q24 according to the manufacturer´s instructions with PyroMark Gold Q24 reagents (Qiagen, Hilden, Germany). Briefly, 10 µl PCR product was incubated with Sepharose beads, binding buffer and H_2_O for 10 minutes on a shaker. Using the Q24 vacuum station, the PCR product-coated beads were washed and then mixed with the pyrosequencing primer. After denaturation and annealing, pyrosequencing was started, filling the cartridge with the amounts of enzyme, substrate and nucleotides indicated and adjusted in the pre-run information. The percentage of methylation of a given CpG position was displayed in the pyrogram of the instrument (Pyromark Q24 Version2.0.7).

If more than one methylation analysis was available for a patient, the mean value of methylation was calculated and used for analysis. Samples from the same patient were run in the same assay once. Results from pyrosequencing runs with control results outside the defined range (mean +/2 SD) were excluded.

### Characterization of the *GHR* Genotype

The full length/exon 3-deleted *GHR* polymorphism was genotyped by PCR (3). A DNA sample from each patient was amplified twice, first with primers G1/G3 amplifying the 935 bp long allele and then with primers G1/G2 leading to the 532 bp long exon 3-deleted allele. In a subsequent agarose gel, the genotype could be determined from the amplified PCR product(s).

### IGF-1 and IGFBP-3 Serum Concentrations

Blood samples were centrifuged immediately after collection, and serum was stored at -70°C until further analysis. IGF-1 was measured with a validated in-house IGF-1 RIA equipped with anti-human IGF-1 antibodies (rabbit) and recombinant human IGF-1 as standard (Mediagnost, Reutlingen, Germany). In this assay, an excess of IGF-II is used to exclude interference by IGFBPs (17).

Serum IGFBP-3 was measured using a validated in-house RIA as described previously (18) with minor modifications. The mean inter-and intra-assay coefficients of the IGF-1 and the IGFBP-3 assays were 8.9 and 7% as well as 10 and 6%, respectively. IGF-1 and IGFBP-3 concentrations were converted to age-related SDS values based on a reference population of healthy German and Danish children with normal height (19).

### Statistical Analysis

Height was transformed into SDS according to Prader et al. (20). Birth length and birth weight were transformed into SDS according to Niklasson et al. (21). Growth response to rhGH was calculated using two different approaches. The first approach was to calculate the difference between pre-treatment height velocity and the height velocity of the first year of treatment (delta height velocity in cm/y). A period of 6 to 12 months before the start of treatment was used to calculate the pre-treatment height velocity. The treatment height velocity included the first 12 months of rhGH treatment.

The second approach was to calculate the standardized growth response using specific regression formulas for each diagnosis (22–24). These prediction models were developed using multiple linear regression analysis fitted by least squares and the REG procedure. The difference between observed and predicted height velocity is reported as studentized residuals, which is the standardized deviation of individual height velocity from the median of a comparable cohort of children. For this specific approach, patients´ clinical data were converted to SDS according to the given prediction formula: height was transformed to SDS according to the Tanner references (25), weight to SDS based on the Freeman references (26), and birth weight and birth length were converted to SDS using the Niklasson references (21).

Statistical analysis was performed using the JMP 15.2 software (SAS Institute Inc., Cary, NC, U.S.). Linear regression analyses were performed using methylation as the independent variable and either delta height velocity or studentized residuals as the dependent variable. In addition, linear regression was also performed using methylation as the independent variable and basal IGF-1 SDS or delta IGF-1 SDS as the dependent variable. Delta IGF-1 SDS was calculated as the difference between serum IGF-1 SDS after 12 months of treatment and IGF-1 SDS at baseline.

The strength of relationships between variables was estimated using Pearson´s correlation coefficient (r). In addition, relationships between outcome parameters and methylation were tested using multiple linear regression including additional variables (GHR genotype, sex). Statistical significance was defined by a p-value ≤ 0.05.

## Results

The characteristics of the patients and their response to treatment are shown in **Table 1**. Patients were relatively young at treatment start for all three diagnoses. The response to rhGH treatment was above average in terms of delta height velocity and studentized residuals. A total of 443 patient samples could be used for genetic and epigenetic analysis.

**Table 1:** Clinical characteristics and GHR polymorphism status of the patients (mean ± SD).

Diagnosis	GHD	SGA short stature	Turner syndrome
n	40	36	16
Female/male; n	11/29	12/24	16/0
Birth length; cm	48.9 ± 4.2	41.5 ± 7.2	48.1 ± 2.8
Birth length; SDS	-0.12 ± 1.18	-2.15 ± 1.46	-0.12 ± 1.17
Birth weight; g	2917 ± 729	1893 ± 794	2867 ± 633
Birth weight; SDS	-0.67 ± 1.16	-2.52 ± 1.06	-0.65 ± 1.08
Target height; cm	172.0 ± 7.7	169.4 ± 7.2	164.0 ± 5.4
Age at start of treatment; y [median (range)]	5.2 (0.3-11.9)	6.0 (0.6-9.8)	5.3 (3.9-11.6)
Bone age at start of treatment; y	4.7 ± 2.2	4.9 ± 2.1	6.5 ± 3.1
Height at start of treatment; SDS	-3.9 ± 1.2	-3.9 ± 0.9	-3.4 ± 0.7
RhGH dose; µg/kg*d	28.4 ± 6.7	39.1 ± 5.6	46.2 ± 3.1
Height 1 y after start of treatment; SDS	-2.7 ± 0.8	-3.0 ± 1.1	-2.8 ± 0.8
Height velocity at start of treatment; cm/y	5.0 ± 1.1	5.5 ± 1.1	5.6 ± 2.2
Height velocity 1 y after start of treatment; cm/y	9.9 ± 1.9	8.9 ± 1.0	9.1 ± 1.5
Delta height velocity; cm/y	4.7 ± 1.5	3.4 ± 1.4	3.6 ± 3.3
IGF-1 at start of treatment; SDS	-4.2 ± 1.9	-1.7 ± 1.5	-1.5 ± 1.1
IGF-1 1 y after start of treatment; SDS	-1.0 ± 1.2	0.7 ± 1.3	1.2 ± 0.7
Delta IGF-1 SDS under treatment; SDS	3.2 ± 1.7	2.4 ± 1.5	2.5 ± 1.2
IGFBP-3 at start of treatment; SDS	-3.2 ± 1.8	-1.6 ± 1.3	-1.8 ± 1.2
IGFBP-3 1 y after start of treatment; SDS	-1.0 ± 1.2	-0.1 ± 1.0	-0.8 ± 0.7
Delta IGFBP-3 SDS under treatment; SDS	2.3 ± 1.9	1.5 ± 1.2	1.0 ± 0.9
Studentized residuals of 1^st^ y prediction	0.53 ± 1.1	1.48 ± 1.2	0.32 ± 1.4
GHR polymorphism; wt-wt/wt-del3/del3-del3; n	24/16/0	16/16/4	6/9/1

For calculation of height velocity, patients aged < 4 y at start of rhGH treatment were excluded (n = 13).

The methylation of the P2 promotor of *IGF1* at positions -137 and +97 could be determined in the blood of 40 children with GHD, 36 children with SGA short stature, and 16 children with Turner syndrome. The mean (SD) values at position -137 were 59.5% (6.6) in GHD, 57.6% (7.2) in SGA short stature, and 64.1% (8.1) in Turner syndrome. The percentages at position +97 were as follows: 17.8% (4.8) in GHD, 18.3% (5.4) in SGA short stature, and 22.6% (4.3) in Turner syndrome.

The haplotypes of the GHR polymorphism were as follows: in GHD wt-wt (60%), wt-del3 (40%) and del3-del3 (0%), in SGA short stature wt-wt (44.5%), wt-del3 (44.5%) and del3-del3 (11%), in Turner syndrome wt-wt (38%), wt-del3 (56%) and del3-del3 (6%). In total, 50% of patients were wt-wt, 45% were wt-del3 and 5% were del3-del3.

**Figure 1:**
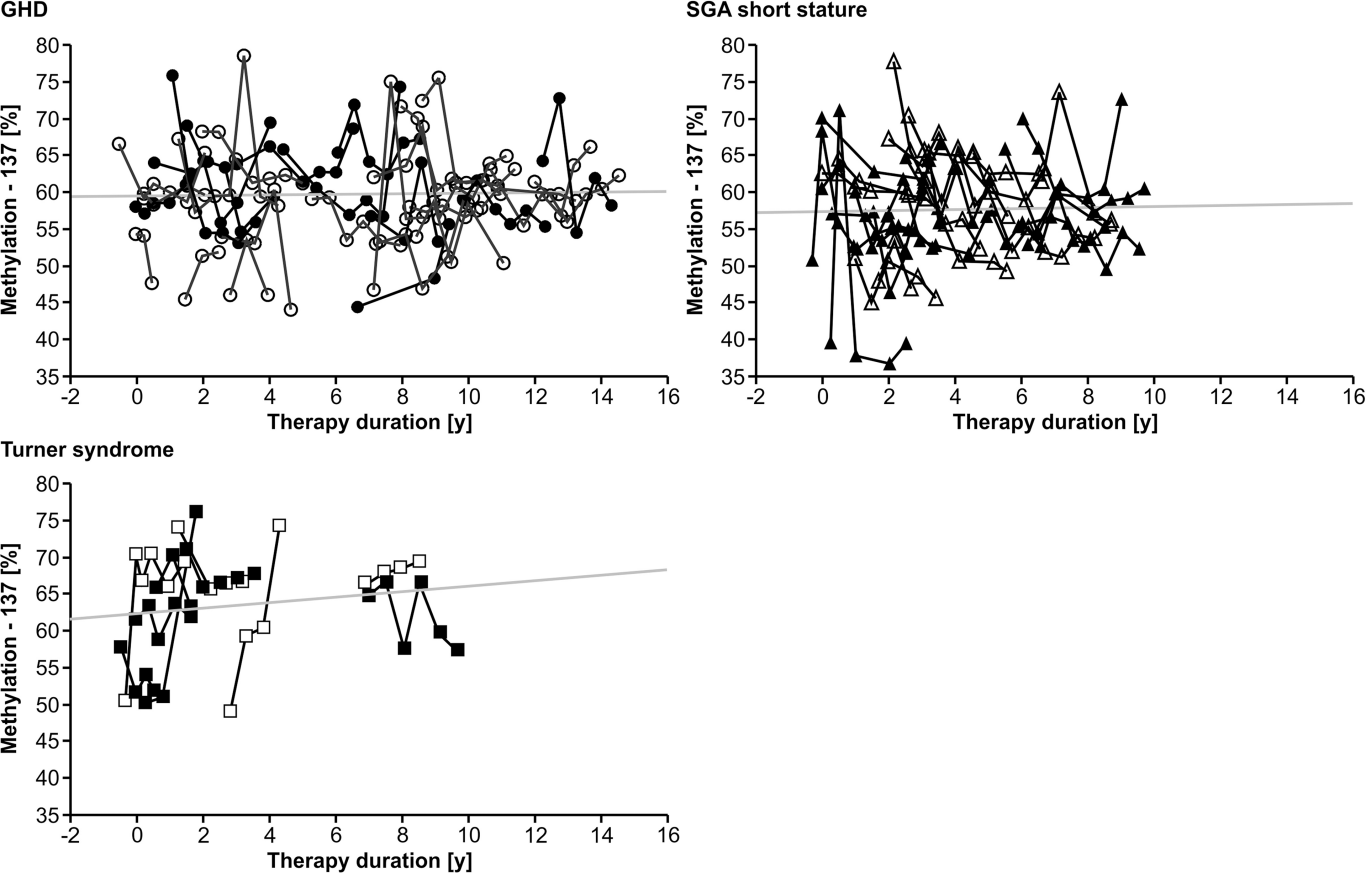
Longitudinal course of *IGF1* P2 methylation at position -137 in blood of children with GHD, SGA short stature and Turner syndrome treated with rhGH. Filled symbols indicate patients with exon3-deleted GHR.

Longitudinal analysis of the methylation over a period of 0.5 years before treatment to 12.0 years during treatment with rhGH showed a variance of 7.1% (-137) and 4.9% (+97), but no specific trend toward lower or higher values over time in any of the three diagnostic groups. There was no unidirectional change in samples from 0, 6 and 12 months during the first year of treatment. There was no longitudinal trend in methylation of the P2 promoter of *IGF1* at position -137 (**Figure 1**). Importantly, the inter-assay variability of the methylation of our control samples was 9.5% (-137) and 6.6% (+97).

**Figure 2:**
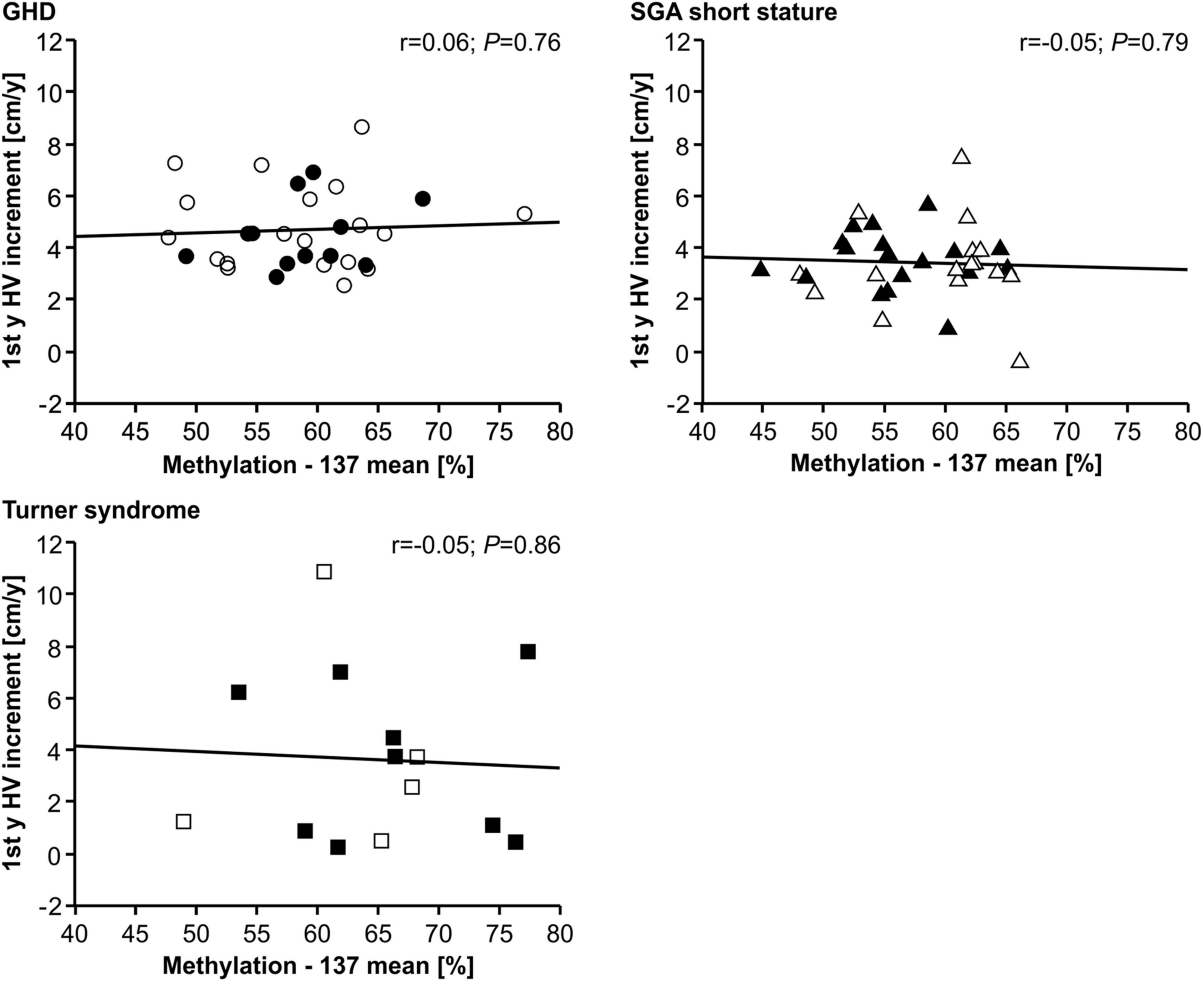
First year height velocity increment (1^st^ y HV increment) and *IGF1* P2 methylation at position -137 in blood of children with GHD, SGA short stature and Turner syndrome treated with rhGH. Filled symbols indicate patients with exon3-deleted GHR allele.

The methylation of the P2 promoter of *IGF1* at position -137 was not correlated with delta height velocity in any of the three diagnostic groups (**Figure 2**). The same result was found for the +97 position (data not shown).

**Figure 3:**
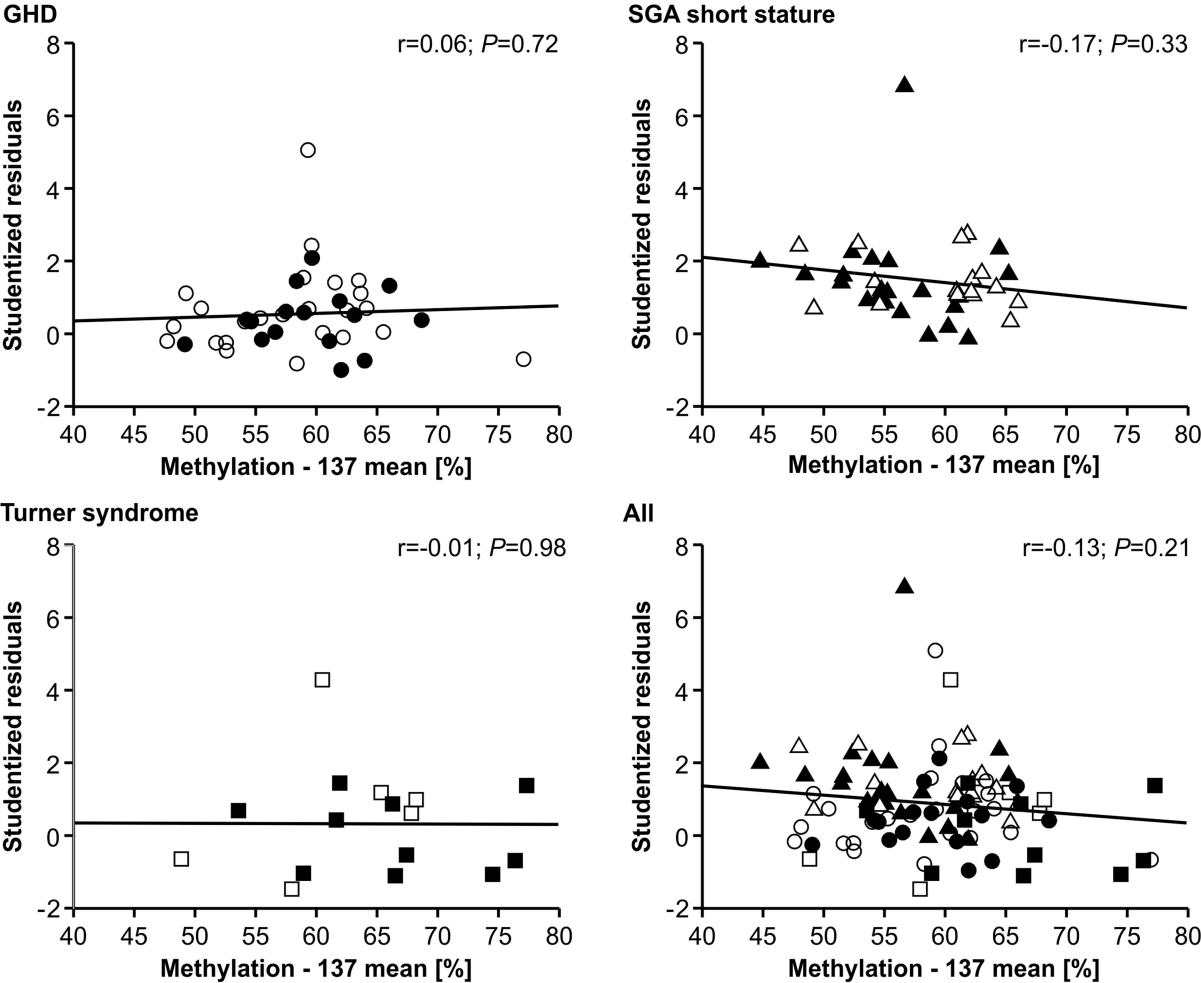
Studentized residuals of first year height prediction and *IGF1* P2 methylation at position -137 in blood of children with GHD, SGA short stature and Turner syndrome treated with rhGH. Filled symbols indicate patients with exon3-deleted GHR allele.

The methylation of the P2 promoter of *IGF1* at position -137 showed no relationship with the studentized residuals of the three diagnostic groups (**Figure 3**). The same result was found for the +97 position (data not shown).

**Figure 4:**
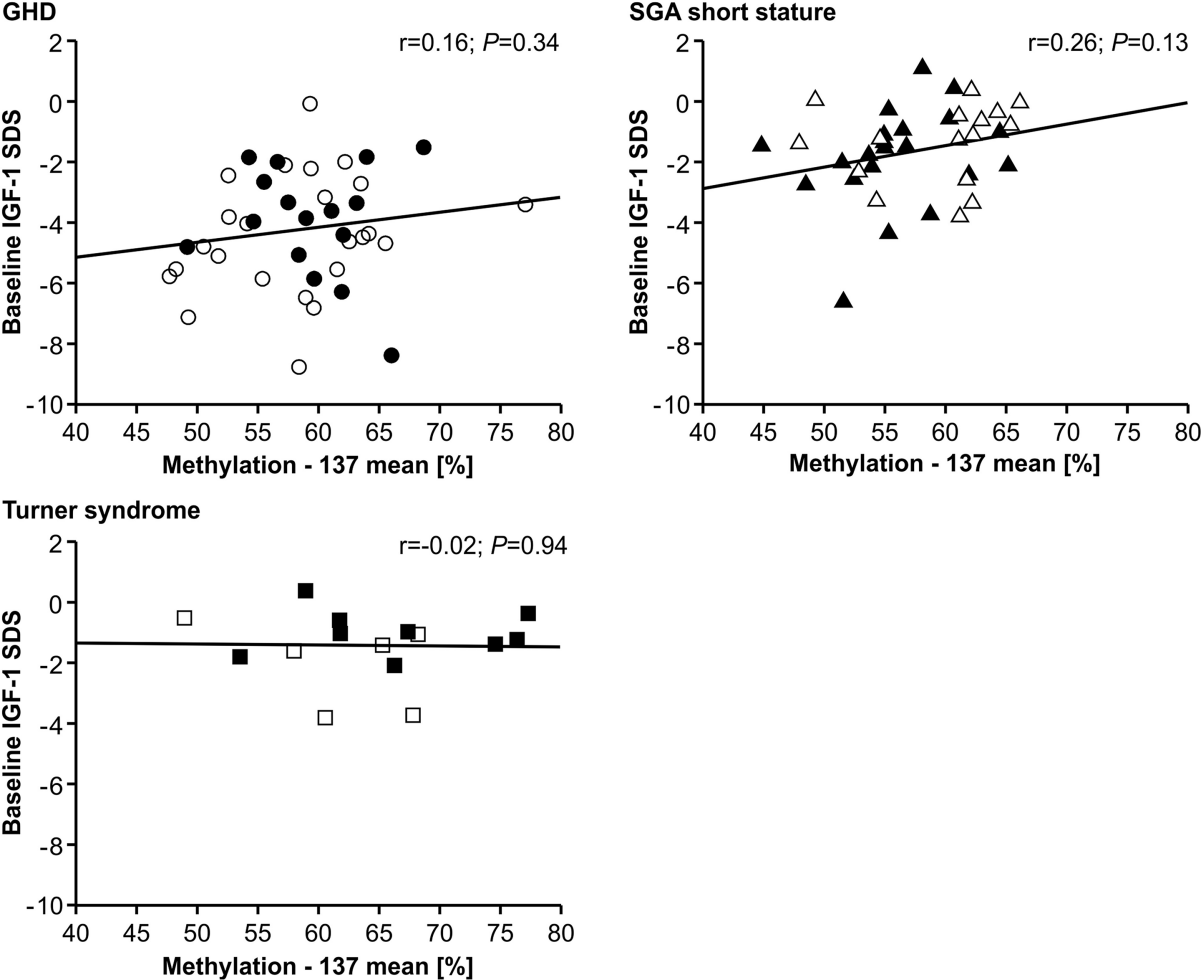
Baseline IGF-1 SDS and *IGF1* P2 methylation at position -137. Filled symbols indicate patients with exon3-deleted GHR allele.

**Figure 5:**
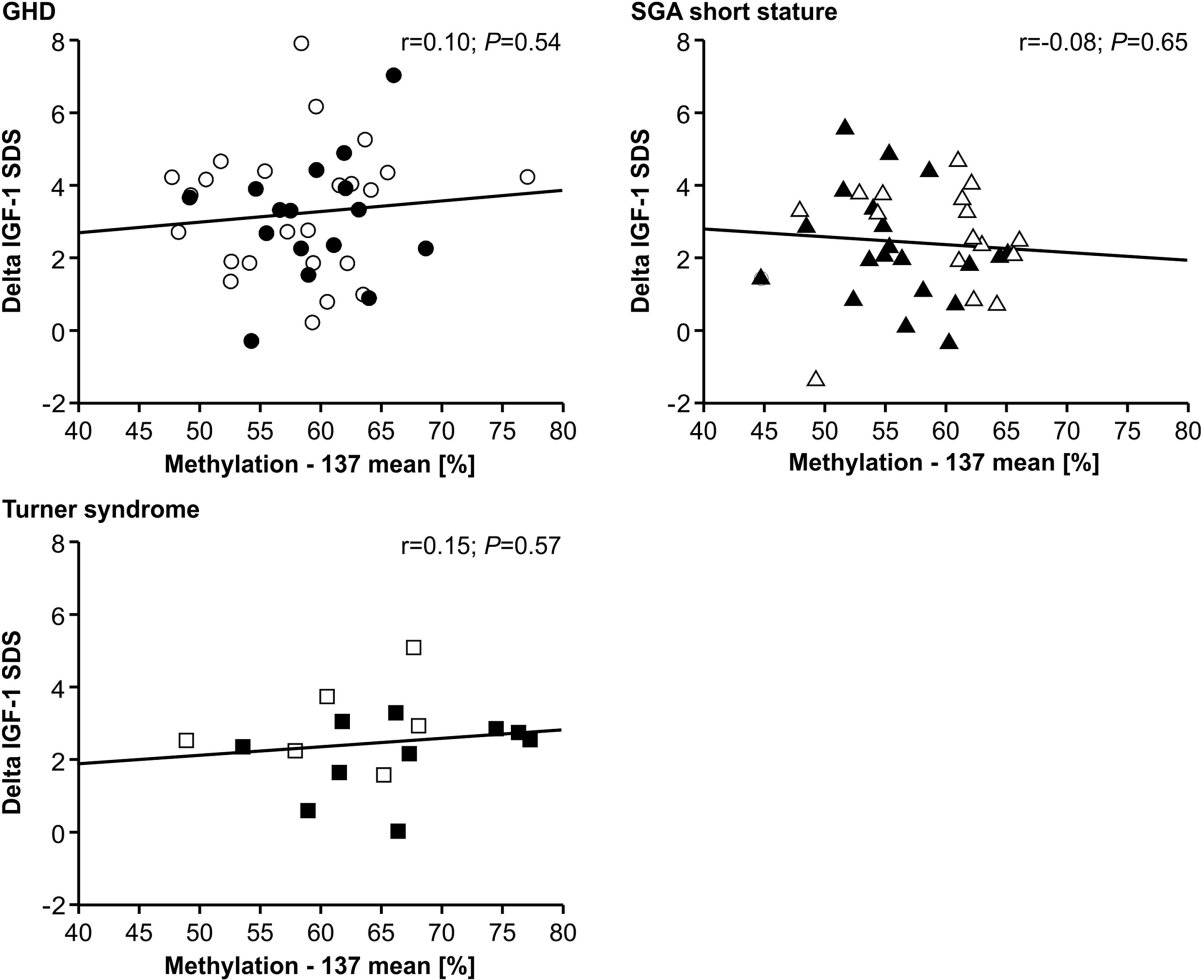
GH-induced increment in IGF-1 SDS (Delta IGF-1 SDS) and *IGF1* P2 methylation at position -137. Filled symbols indicate patients with exon3-deleted GHR allele.

The IGF-1 concentrations at baseline and their increase from baseline to 12 months of treatment (delta SDS) also did not correlate to the P2 promoter methylation (**Figures 4** and **5**).

Multiple linear regression analysis including the GHR genotype (exon 3 deletion versus no exon 3 deletion) did not show any relationship between outcome parameters and the examined genetic characteristics (data not shown.). In addition, the GHR genotype did not correlate to IGF-1 serum concentration or GH responsiveness (data not shown).

## Discussion

Methylation of the *IGF1* promoter P2 at two specific CG-sites was measured in the blood of children with GHD, SGA short stature and Turner syndrome treated with rhGH. Promoter methylation did not correlate with first-year response in terms of height velocity increment or studentized residuals based on a prediction model at either position -137 or position +97. Furthermore, baseline IGF-1 serum concentrations and increases during rhGH treatment did not correlate with *IGF1* P2 promoter methylation. Our analysis is in contradiction with previous reports from Ouni and colleagues that reported such correlations observed in children with low-normal stature or ISS treated with very high doses of rhGH (11).

Ouni et al. used the same source, peripheral blood leucocytes, for epigenetic analysis. In contrast to this study, blood was collected only before the start of rhGH treatment by the French group, whereas 94% of our samples were collected during rhGH treatment. However, the intraindividual changes of promoter methylation observed in our study were small after the start of rhGH treatment and showed no trend. The pyrosequencing protocols were identical. The French group observed a mean of 49% methylation at the -137 site of promoter P2, and 17% methylation at the +97 site. Here, the methylation values were 60% at -137 and 19% at +97, approximately 20% higher.

Ouni et al. treated 136 children with ISS (or low-normal stature) with a high pharmacological rhGH dose of 67 µg/kg*day and observed an inverse relationship between first-year increment of height velocity and the methylation at the *IGF1* P2 methylation site -137 in blood cells (11). They calculated that 25% of the variability of the height velocity increment could be explained by the methylation at this *IGF1* promoter site. Our study cohort did not include children with ISS, but GHD, SGA short stature and Turner syndrome. The height velocity increments were measured in the same way. Our patients were by 2.5 years younger and the sample size was smaller than in the French studies. The rhGH doses here ranged from 20 to 60 µg/kg*day, which was lower than in the French study. Although the studied children and the treatment dose were different, one would expect at least a trend in the same direction if the reported correlation is not specific for ISS and for very high doses of rhGH. Moreover, our analysis of responsiveness including relevant outcome parameters of individual patients (studentized residuals of prediction) showed no lower response to rhGH in patients with a higher methylation of *IGF1* P2 promoter sites.

Similarly, Ouni et al. reported an inverse correlation of basal serum IGF-1 concentration with the methylation at -137 of the *IGF1* P2 promoter in a mixed group of children with normal or idiopathic short stature (14). Moreover, the short-term IGF-1 increase in the IGF-1 generation test (SDS and concentration) was shown to be higher in children with lower methylation of the *IGF1* promoter (11, 15). We analyzed baseline IGF-1 and the increment after 12 months of rhGH treatment and found no correlation with the methylation status of the *IGF1* promoter. In conclusion, there are significant differences in study conditions between the French studies and our study, including different rhGH dose, different diagnoses, and different timing of IGF-1 observation, which may explain the conflicting results.

In a second part of the study, we examined the stability of the methylation marks of the *IGF1* P2 promoter in blood cells over time during rhGH treatment. The data suggest good stability and no specific change over time. However, we observed intraindividual variation in long-term results, which may possibly reflect changes in the number of different leucocyte populations in blood specimens.

In conclusion, we could not confirm that *IGF1* P2 promoter methylation is an important epigenetic locus for GH responsiveness in children with GHD, SGA short stature and Turner syndrome. This nonconfirmatory study will hopefully motivate other research groups to publish their findings or to perform new analyses, because more data are needed for a definitive judgement in this field of pharmacogenetics.

## Data Availability Statement

The original contributions presented in the study are included in the article/supplementary material. Further inquiries can be directed to the corresponding author.

## Ethics Statement

The studies involving human participants were reviewed and approved by Ethics Committe of the Medical Faculty at the University of Tübingen. Written informed consent to participate in this study was provided by the participants’ legal guardian/next of kin.

## Author Contributions

GBi and AA contributed to conception and design of the study. RS, AA and DII organized the database. AA, DII, CU, KW, SP and VN performed or supervised the (epi-)genetic analyses. AA and GBl performed the statistical analysis. AA and GBi wrote the first draft of the manuscript. All authors contributed to manuscript revision, read, and approved the submitted version.

## Funding

The research project was supported by a grant from Novo Nordisk Pharma GmbH, Mainz, Germany (Investigator Sponsored Study U1111-1248-5097). The funder was not involved in the study design, collection, analysis, interpretation of data, the writing of this article or the decision to submit it for publication. All authors declare no other competing interests. DII was supported by DAAD funding program “Research Stays for University Academics and Scientists” (Number 57552334, year 2021).

## Acknowledgments

Technical support of pyrosequencing by Mara Thomas, Nora Knoblich and Ariane Wiegand was greatly appreciated. We are thankful to Joachim Woelfle for fruitful discussion during designing the study.

## Conflict of Interest

The authors declare that the research was conducted in the absence of any commercial or financial relationships that could be construed as a potential conflict of interest.

## Publisher’s Note

All claims expressed in this article are solely those of the authors and do not necessarily represent those of their affiliated organizations, or those of the publisher, the editors and the reviewers. Any product that may be evaluated in this article, or claim that may be made by its manufacturer, is not guaranteed or endorsed by the publisher.

## References

[1] Ranke MB Wit JM. Growth Hormone - Past, Present and Future. Nat Rev Endocrinol (2018) 14:285–300. doi: 10.1038/nrendo.2018.22 29546874

[2] Stevens A De Leonibus C Whatmore A Hanson D Murray P Chatelain P. Pharmacogenomics Related to Growth Disorders. Horm Res Paediatr (2013) 80:477–90. doi: 10.1159/000355658 24296333

[3] Pantel J Machinis K Sobrier ML Duquesnoy P Goossens M Amselem S. Species-Specific Alternative Splice Mimicry at the Growth Hormone Receptor Locus Revealed by the Lineage of Retroelements During Primate Evolution. J Biol Chem (2000) 275:18664–9. doi: 10.1074/jbc.M001615200 10764769

[4] Dos Santos C Essioux L Teinturier C Tauber M Goffin V Bougneres P. A Common Polymorphism of the Growth Hormone Receptor Is Associated With Increased Responsiveness to Growth Hormone. Nat Genet (2004) 36:720–4. doi: 10.1038/ng1379 15208626

[5] Binder G Baur F Schweizer R Ranke MB. The D3-Growth Hormone (GH) Receptor Polymorphism Is Associated With Increased Responsiveness to GH in Turner Syndrome and Short Small-for-Gestational-Age Children. J Clin Endocrinol Metab (2006) 91:659–64. doi: 10.1210/jc.2005-1581 16291706

[6] Wassenaar MJ Dekkers OM Pereira AM Wit JM Smit JW Biermasz NR. Impact of the Exon 3-Deleted Growth Hormone (GH) Receptor Polymorphism on Baseline Height and the Growth Response to Recombinant Human GH Therapy in GH-Deficient (GHD) and Non-GHD Children With Short Stature: A Systematic Review and Meta-Analysis. J Clin Endocrinol Metab (2009) 94:3721–30. doi: 10.1210/jc.2009-0425 19584188

[7] Renehan AG Solomon M Zwahlen M Morjaria R Whatmore A Audi L. Growth Hormone Receptor Polymorphism and Growth Hormone Therapy Response in Children: A Bayesian Meta-Analysis. Am J Epidemiol (2012) 175:867–77. doi: 10.1093/aje/kwr408 22494952

[8] Deal C Ma J Wilkin F Paquette J Rozen F Ge B. Novel Promoter Polymorphism in Insulin-Like Growth Factor-Binding Protein-3: Correlation With Serum Levels and Interaction With Known Regulators. J Clin Endocrinol Metab (2001) 86:1274–80. doi: 10.1210/jc.86.3.1274 11238520

[9] Braz AF Costalonga EF Montenegro LR Trarbach EB Antonini SR Malaquias AC. The Interactive Effect of GHR-Exon 3 and -202 a/C IGFBP3 Polymorphisms on Rhgh Responsiveness and Treatment Outcomes in Patients With Turner Syndrome. J Clin Endocrinol Metab (2012) 97:E671–7. doi: 10.1210/jc.2011-2521 22278433

[10] Costalonga EF Antonini SR Guerra G Jr Coletta RR Franca MM Braz AF. Growth Hormone Pharmacogenetics: The Interactive Effect of a Microsatellite in the IGF1 Promoter Region With the GHR-Exon 3 and -202 a/C IGFBP3 Variants on Treatment Outcomes of Children With Severe GH Deficiency. Pharmacogenomics J (2012) 12:439–45. doi: 10.1038/tpj.2011.13 21468024

[11] Ouni M Belot MP Castell AL Fradin D and Bougneres P. The P2 Promoter of the IGF1 Gene Is a Major Epigenetic Locus for GH Responsiveness. Pharmacogenomics J (2016) 16(11):102–6. doi: 10.1038/tpj.2015.26 PMC4746489 25869012

[12] Smith ZD Meissner A. DNA Methylation: Roles in Mammalian Development. Nat Rev Genet (2013) 14(3):204–20. doi: 10.1038/nrg3354 23400093

[13] Alvarez-Nava F Lanes R. GH/IGF-1 Signaling and Current Knowledge of Epigenetics; A Review and Considerations on Possible Therapeutic Options. Int J Mol Sci (2017) 18(10):1624. doi: 10.3390/ijms18101624 PMC5666699 28981462

[14] Ouni M Gunes Y Belot MP Castell AL Fradin D Bougneres P. The IGF1 P2 Promoter Is an Epigenetic QTL for Circulating IGF1 and Human Growth. Clin Epigenet (2015) 7:22. doi: 10.1186/s13148-015-0062-8 PMC4363053 25789079

[15] Ouni M Castell AL Linglart A Bougneres P. Genetic and Epigenetic Modulation of Growth Hormone Sensitivity Studied With the IGF-1 Generation Test. J Clin Endocrinol Metab (2015) 100:E919–25. doi: 10.1210/jc.2015-1413 PMC4454803 25835289

[16] Greulich WW Pyle S. Radiograph Atlas of Skeletal Development of the Hand and Wrist. 2nd Ed. California: Stanford University Press (1959).

[17] Blum WF Breier BH. Radioimmunoassays for IGFs and IGFBPs. Growth Regul (1994) 4 Suppl 1:11–9. 7515738

[18] Blum WF Ranke MB Kietzmann K Gauggel E Zeisel HJ Bierich JR. A Specific Radioimmunoassay for the Growth Hormone (GH)-Dependent Somatomedin-Binding Protein: Its Use for Diagnosis of GH Deficiency. J Clin Endocrinol Metab (1990) 70:1292–8. doi: 10.1210/jcem-70-5-1292 1692331

[19] Blum WF Schweizer R. Insulin-Like Growth Factors and Their Binding Proteins. In: Ranke, editor. Diagnostics of Endocrine Functionin Children and Adolescents. Basel: Karger (2003). p. 166–99.

[20] Prader A Largo RH Molinari L Issler C. Physical Growth of Swiss Children From Birth to 20 Years of Age. First Zurich Longitudinal Study of Growth and Development. Helv Paediatr Acta Suppl (1989) 52:1–125. 2737921

[21] Niklasson A Ericson A Fryer JG Karlberg J Lawrence C Karlberg P. An Update of the Swedish Reference Standards for Weight, Length and Head Circumference at Birth for Given Gestational Age (1977-1981). Acta Paediatr Scand (1991) 80:756–62. doi: 10.1111/j.1651-2227.1991.tb11945.x 1957592

[22] Ranke MB Lindberg A Chatelain P Wilton P Cutfield W Albertsson-Wikland K. Derivation and Validation of a Mathematical Model for Predicting the Response to Exogenous Recombinant Human Growth Hormone (GH) in Prepubertal Children With Idiopathic GH Deficiency. KIGS International Board. Kabi Pharmacia International Growth Study. J Clin Endocrinol Metab (1999) 84:1174–83. doi: 10.1210/jcem.84.4.5634 10199749

[23] Ranke MB Lindberg A Chatelain P Wilton P Cutfield W Albertsson-Wikland K. Kabi International Growth Study. Prediction of Long-Term Response to Recombinant Human Growth Hormone in Turner Syndrome: Development and Validation of Mathematical Models. KIGS International Board. Kabi International Growth Study. J Clin Endocrinol Metab (2000) 85:4212–8. doi: 10.1210/jcem.85.11.6976 11095456

[24] Ranke MB Lindberg A Cowell CT Wikland KA Reiter EO Wilton P. Prediction of Response to Growth Hormone Treatment in Short Children Born Small for Gestational Age: Analysis of Data From KIGS (Pharmacia International Growth Database). J Clin Endocrinol Metab (2003) 88:125–31. doi: 10.1210/jc.2002-020867 12519840

[25] Tanner JM Whitehouse RH. Clinical Longitudinal Standards for Height, Weight, Height Velocity, Weight Velocity, and Stages of Puberty. Arch Dis Child (1976) 51:170–9. doi: 10.1136/adc.51.3.170 PMC1545912 952550

[26] Freeman JV Cole TJ Chinn S Jones PR White EM Preece MA. Cross Sectional Stature and Weight Reference Curves for the UK. Arch Dis Child (1995) 73:17–24. doi: 10.1136/adc.73.1.17 7639543 PMC1511167

