# Corrigendum: GH responsiveness is not correlated to *IGF1* P2 promoter methylation in children with Turner syndrome, GHD and SGA short stature

**DOI:** 10.3389/fendo.2022.1020804

**Published:** 2022-09-27

**Authors:** Anja Apel, Daniel I. Iliev, Christina Urban, Karin Weber, Roland Schweizer, Gunnar Blumenstock, Sarah Pasche, Vanessa Nieratschker, Gerhard Binder

**Affiliations:** ^1^ Pediatric Endocrinology, University Children`s Hospital Tübingen, Tübingen, Germany; ^2^ Department of Clinical Epidemiology and Applied Biometry, University of Tübingen, Tübingen, Germany; ^3^ Department of Psychiatry and Psychotherapy, Tübingen Center for Mental Health, University Hospital Tübingen, Tübingen, Germany

**Keywords:** IGF-1, promotor methylation, GH response, prediction, short stature, GH treatment

In the published article, there was an error in **Table 1**. The values of height SDS at and after start of treatment were wrong. The corrected **Table 1** and its caption "Clinical characteristics and GHR polymorphism status of the patients (mean ± SD)" appear below.

**Table 1:** Clinical characteristics and GHR polymorphism status of the patients (mean ± SD).

Diagnosis	GHD	SGA short stature	Turner syndrome
n	40	36	16
Female/male; n	11/29	12/24	16/0
Birth length; cm	48.9 ± 4.2	41.5 ± 7.2	48.1 ± 2.8
Birth length; SDS	−0.12 ± 1.18	−2.15 ± 1.46	−0.12 ± 1.17
Birth weight; g	2,917 ± 729	1,893 ± 794	2,867 ± 633
Birth weight; SDS	−0.67 ± 1.16	−2.52 ± 1.06	−0.65 ± 1.08
Target height; cm	172.0 ± 7.7	169.4 ± 7.2	164.0 ± 5.4
Age at start of treatment; y (median (range))	5.2 (0.3–11.9)	6.0 (0.6–9.8)	5.3 (3.9–11.6)
Bone age at start of treatment; y	4.7 ± 2.2	4.9 ± 2.1	6.5 ± 3.1
Height at start of treatment; SDS	−3.9 ± 1.2	−3.9 ± 0.9	−3.4 ± 0.7
RhGH dose; µg/kg*d	28.4 ± 6.7	39.1 ± 5.6	46.2 ± 3.1
Height 1 y after start of treatment; SDS	−2.7 ± 0.8	−3.0 ± 1.1	−2.8 ± 0.8
Height velocity at start of treatment; cm/y	5.0 ± 1.1	5.5 ± 1.1	5.6 ± 2.2
Height velocity 1 y after start of treatment; cm/y	9.9 ± 1.9	8.9 ± 1.0	9.1 ± 1.5
Delta height velocity; cm/y	4.7 ± 1.5	3.4 ± 1.4	3.6 ± 3.3
IGF-1 at start of treatment; SDS	−4.2 ± 1.9	−1.7 ± 1.5	−1.5 ± 1.1
IGF-1 1 y after start of treatment; SDS	−1.0 ± 1.2	0.7 ± 1.3	1.2 ± 0.7
Delta IGF-1 SDS under treatment; SDS	3.2 ± 1.7	2.4 ± 1.5	2.5 ± 1.2
IGFBP-3 at start of treatment; SDS	−3.2 ± 1.8	−1.6 ± 1.3	−1.8 ± 1.2
IGFBP-3 1 y after start of treatment; SDS	−1.0 ± 1.2	−0.1 ± 1.0	−0.8 ± 0.7
Delta IGFBP-3 SDS under treatment; SDS	2.3 ± 1.9	1.5 ± 1.2	1.0 ± 0.9
Studentized residuals of 1st y prediction	0.53 ± 1.1	1.48 ± 1.2	0.32 ± 1.4
GHR polymorphism; wt-wt/wt-del3/del3-del3; n	24/16/0	16/16/4	6/9/1

For calculation of height velocity, patients aged <4 y at start of rhGH treatment were excluded (n = 13).

The authors apologize for this error and state that this does not change the scientific conclusions of the article in any way. The original article has been updated.

## Publisher’s note

All claims expressed in this article are solely those of the authors and do not necessarily represent those of their affiliated organizations, or those of the publisher, the editors and the reviewers. Any product that may be evaluated in this article, or claim that may be made by its manufacturer, is not guaranteed or endorsed by the publisher.

